# 874. Diagnostic Stewardship of Gastrointestinal Multiplex PCR Panels in Hospitalized Children

**DOI:** 10.1093/ofid/ofac492.067

**Published:** 2022-12-15

**Authors:** Jillian Cotter, Claire Stokes, Suhong Tong, Samuel R Dominguez

**Affiliations:** University of Colorado, Aurora, Colorado; Children’s Healthcare of Atlanta/Emory University, Atlanta, Georgia; University of Colorado, Aurora, Colorado; University of Colorado, Aurora, Colorado

## Abstract

**Background:**

The introduction of multiplex gastrointestinal panels (GIP) led to increased rates of children with stool testing, high rates of negative results, and no change in clinical outcomes for most children. We aimed to reduce overall stool testing and stool testing with negative results by 20%.

**Methods:**

We engaged stakeholders and implemented multi-modal interventions including 1) education and development of a clinical pathway to guide testing, and 2) electronic medical record (EMR)-based testing changes including built in stool testing restrictions with optional approval process (Table 1), and quantification of stool caliber in the EMR by nursing using the validated Bristol Stool Scale (BSS). We included all hospitalized children who had stool testing performed at a large children’s hospital over 30 months (2018–2020). Outcomes included the rate of stool tests and negative stool results per 10,000 patient-days. We evaluated rates of BSS documentation and GIPs performed for children hospitalized for > 96 hours as process measures. Balancing measures included the percent of ordered tests that were restricted, and percent of initially restricted tests that were approved. We compared the rate of actionable results (bacteria or parasite identified) between initially restricted but ultimately approved tests and non-restricted tests.

**Results:**

There were 2,001 tests performed for 1,982 encounters (Table 2). After interventions, we found special cause variation with a 29% sustained decrease in the rate of stool testing and a 28% decrease in the rate of negative stool results (Figures 1,2). The rate of use of BSS was 81% and was sustained. There was a 33% decrease in the number of GIPs performed for children hospitalized for >96 hours. 34% of ordered stool tests per month were restricted, and this did not change over time. 56% of initially restricted tests were ultimately approved with no change over time, and these tests had a lower rate of actionable results compared to non-restricted tests (25 vs 34%, p=0.02).

**Conclusion:**

Education coupled with EMR-based testing restrictions resulted in reduced rates of overall stool testing and low-value stool testing. Sustained ordering of restricted tests over time suggests that EMR-decision support is needed and likely cannot be removed.

**Disclosures:**

**Samuel R. Dominguez, MD PhD**, BioFire Diagnostics: Advisor/Consultant|BioFire Diagnostics: Grant/Research Support|Karius: Advisor/Consultant|Pfizer: Grant/Research Support.

**Figure:**
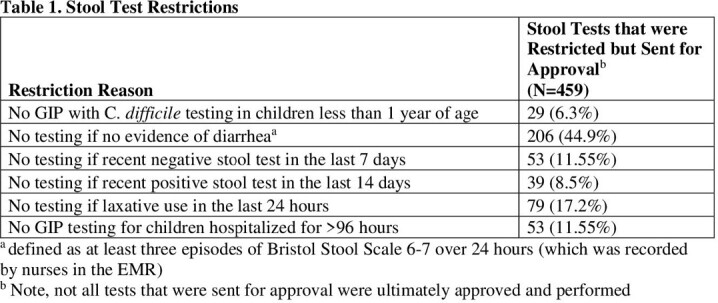

**Figure:**
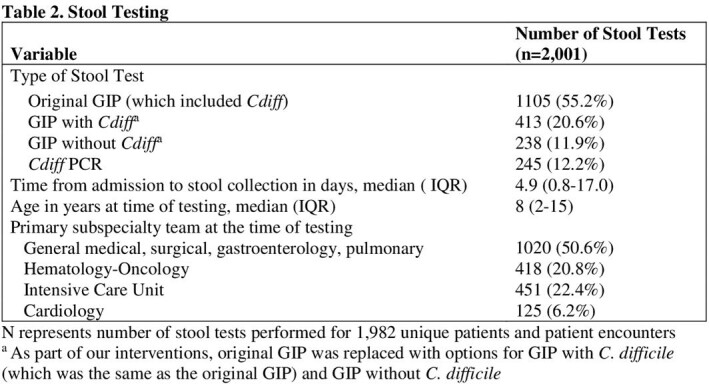

**Figure 1.**
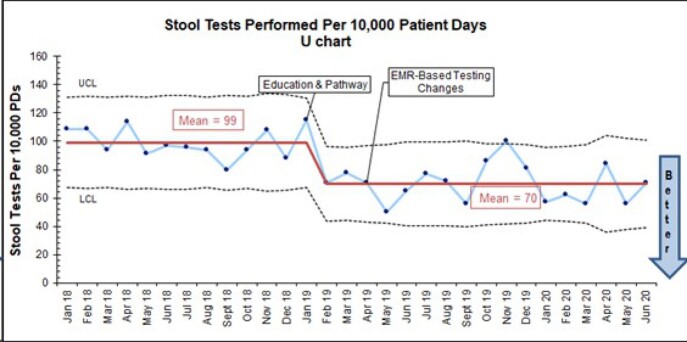
Stool Tests Performed Per 10,000 Patient-Days

**Figure 2.**
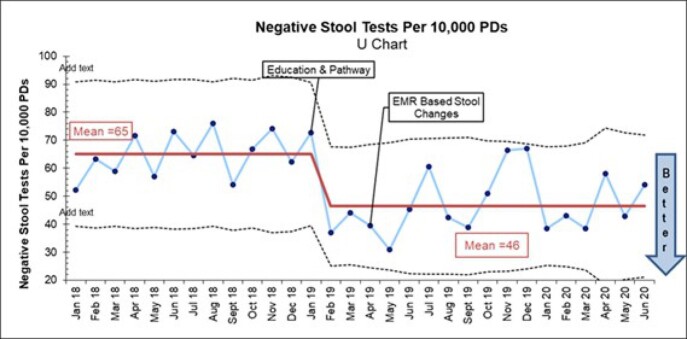
Stool Tests with Negative Results Per 10,000 Patient-Days

